# Animal Signatures

**Published:** 1858-03

**Authors:** 


AN INDIAN DOCTRINE OF ANIMAL SIGNATURES.—Tigers' flesh is a favorite specific amongst the wandering tribes of the Decan, and large quantities of it are consumed in all diseases where medicine of a healing nature is required. It is also believed to predispose to anger, and swallowed from a motive analogous to that of the running footman who ate hare to make him fleet. There is always sure to be in some corner of the wallet a small bottle or gourd full of tigers'-fat, a sovereign remedy against all the infirmities of old age. The Weyds also carry quantities of jackals'-skins, which they sell to persons who believe them to be very efficacious in curing rheumatism.—*Chow Chow*, by Viscountess Falkland. (*Lancet*, Dec. 19, 1857.)

